# The abundance of the ARL2 GTPase and its GAP, ELMOD2, at mitochondria are modulated by the fusogenic activity of mitofusins and stressors

**DOI:** 10.1371/journal.pone.0175164

**Published:** 2017-04-05

**Authors:** Laura E. Newman, Cara R. Schiavon, Chengjing Zhou, Richard A. Kahn

**Affiliations:** Department of Biochemistry, Emory University School of Medicine, Atlanta, Georgia, United States of America; Beatson Institute for Cancer Research, UNITED KINGDOM

## Abstract

Mitochondria are essential, dynamic organelles that respond to a number of stressors with changes in morphology that are linked to several mitochondrial functions, though the mechanisms involved are poorly understood. We show that the levels of the regulatory GTPase ARL2 and its GAP, ELMOD2, are specifically increased at mitochondria in immortalized mouse embryo fibroblasts deleted for Mitofusin 2 (MFN2), but not MFN1. Elevated ARL2 and ELMOD2 in MEFs deleted for MFN2 could be reversed by re-introduction of MFN2, but only when the mitochondrial fragmentation in these MEFs was also reversed, demonstrating that reversal of elevated ARL2 and ELMOD2 requires the fusogenic activity of MFN2. Other stressors with links to mitochondrial morphology were investigated and several, including glucose or serum deprivation, also caused increases in ARL2 and ELMOD2. In contrast, a number of pharmacological inhibitors of energy metabolism caused increases in ARL2 without affecting ELMOD2 levels. Together we interpret these data as evidence of two ARL2-sensitive pathways in mitochondria, one affecting ATP levels that is independent of ELMOD2 and the other leading to mitochondrial fusion involving MFN2 that does involve ELMOD2.

## Introduction

Mitochondria are essential organelles that are hubs for several important cellular functions, including ATP production, lipid metabolism, calcium regulation, and apoptosis. This diversity of essential functions is accompanied by diversity in morphology as mitochondria are highly dynamic organelles that can range in size and shape from many small spheres to one large inter-connected network. The linkages between function and morphology must be sensitive to cues coming from other parts of the cell [1–3]. Mitochondrial morphology is the result of a balance between fission and fusion, which are mediated by four large dynamin-related GTPases. Mitochondrial fission is mediated by DRP1 [4, 5], while fusion is controlled by three GTPases: MFN1 and MFN2 regulate outer membrane fusion [6, 7], and OPA1 promotes inner membrane fusion [8]. Mitochondria elongate during several types of stress as a result of increased fusion [9, 10] and also elongate during starvation, protecting them from autophagy [11, 12]. To date, only a handful of proteins have been shown to regulate either mitofusins or OPA1, and how these regulators promote fusion under stress is an area of ongoing research.

We recently discovered that ARL2 plays a role in the regulation of mitochondrial fusion (Newman et al., submitted). ARL2, a ~20 kDa member of the ARF family of regulatory GTPases, is very highly conserved throughout eukaryotic evolution, ubiquitously expressed, predicted to be present in the last eukaryotic common ancestor [13], and is essential in eukaryotes [14–16]. ARL2 plays essential roles in the biogenesis of tubulin and in microtubule dynamics, as well as traffic of farnesylated proteins [17–24]. Only later was ARL2 found to also localize specifically to mitochondria, where it also plays essential roles. ARL2 siRNA causes mitochondrial fragmentation, a loss in plus-end directed mitochondrial motility, and a dramatic (~50%) loss in cellular ATP [25]. ARL2 also regulates mitochondrial fusion from the IMS, acting upstream to increase fusion requiring either MFN1 or MFN2 (Newman, et al., submitted).

We also identified and purified the ARL2 GAP, ELMOD2, and found that it too localizes to mitochondria [26]. ELMOD2 siRNA results in fragmentation and perinuclear clustering but has no effect on ATP levels. As a result, we currently model ARL2 as having at least two distinguishable actions in mitochondria: one leading to regulation of ATP production and a separate one that involves ELMOD2 and impacting fusion and motility.

Mitochondria play crucial roles in several essential cellular processes and must be sensitive to inputs from different parts of the cell to maintain homeostasis or respond to a changing environment. With the identification of a regulatory GTPase and an effector/GAP implicated as regulators of fusion and motility, we sought to examine whether they may be responsive to stressors that are known to impact mitochondrial morphology and functions. Here, we show that the levels of mitochondrial ARL2 and ELMOD2 are highly sensitive to mitochondrial stress and changes in the levels of MFN2. These results further the model that ARL2 and ELMOD2 are components in a system of communication between mitochondria and other parts of the cell.

## Results

### Mitochondrial ARL2 staining is sensitive to re-plating and cell density

Studies of ARL2 began in the 1990’s with a focus on its role in microtubules as a result of data from genetic studies in several model organisms [14–16, 27–29]. In contrast, studies in our lab during and since that period have consistently pointed to mitochondria as an important site of action for ARL2 [25, 30, 31]. Characterization of our specific rabbit polyclonal antibody directed against ARL2 allowed immunofluorescent and immunoblotting evidence of the existence of a mitochondrial pool of ARL2, estimated at ~5% of total cellular ARL2 [30]. Throughout our studies we have noted that the intensity of mitochondrial staining of ARL2 varied between experiments and cell lines. With our long term goal of understanding the mechanisms of both ARL2 regulation of mitochondrial fusion (Newman et al., submitted) and actions in other parts of the cell, we sought to better understand the sources of variation in staining of mitochondrial ARL2. Systematic and careful analysis of imaging data allowed us to identify a number of conditions that contribute to variations in the intensity of imaging data for ARL2 in mitochondria. These are described below and provide further support for our conclusion that ARL2 and its GAP, ELMOD2, regulate mitofusins and mitochondrial fusion and are themselves regulated in abundance at mitochondria.

**Fig 1:**
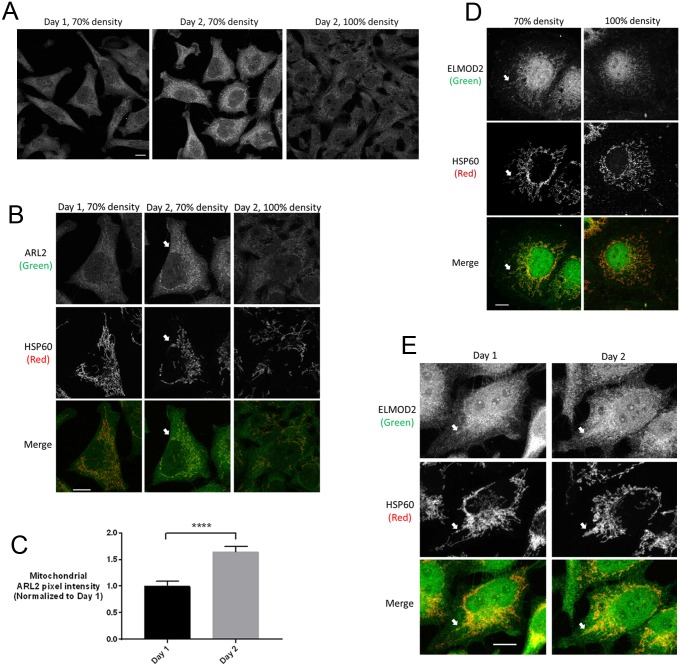
Mitochondrial staining of ARL2 and ELMOD2 vary in intensity with cell density and days after plating. (**A**) HeLa cells were fixed at ~70% confluence either one (left) or two days (middle) after plating, or seeded at higher density and fixed at 100% confluence at 2 days (right). Cells were fixed and stained for ARL2 and HSP60, as described under Methods. (**B**) One cell from each field in panel A was selected and enlarged. ARL2 (top) and HSP60 staining (middle) are shown separately, with merged images on the bottom. Images are single planes acquired by confocal microscopy. Images in A and B are single planes acquired by confocal microscopy; scale bars = 10 μm. (**C**) Mitochondrial ARL2 pixel intensity was quantified in cells plated one or two days, as described under Materials and Methods. For each condition, 10 cells were analyzed, and the increase in ARL2 signal in Day 2 cells was statistically significant (p<0.0001). (**D**) COS7 cells at either 70% or 100% density were fixed 2 days after plating and stained for ELMOD2 and HSP60. (**E**) HeLa cells were fixed at 70% confluence and stained for ELMOD2 and HSP60. ELMOD2 (top) and HSP60 staining (middle) are shown, with merged images on the bottom. Images are z stack projections, and scale bars = 10 μm. Nuclear staining of ELMOD2 is non-specific, as described in the text.

**Fig 2:**
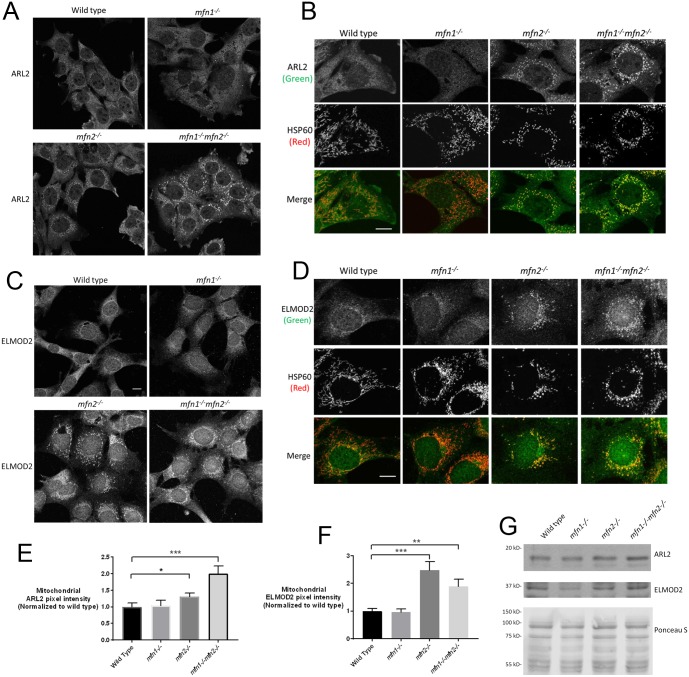
Mitochondrial ARL2 and ELMOD2 are increased in *mfn2*^*-/-*^ and *mfn1*^*-/-*^*mfn2*^*-/-*^ MEFs. Wild type, *mfn1*^*-/-*^, *mfn2*^*-/-*^, or *mfn1*^*-/-*^*mfn2*^*-/-*^ MEFs were fixed (50% density) one day after plating and stained for ARL2 or HSP60. (**A**) ARL2 is shown for each line. (**B**) Single cells stained for ARL2 (top) and HSP60 (middle) are shown and merged (bottom). Single z planes are shown. (**C**) Same as A, except cells were stained for ELMOD2. Z stack projections are shown. (**D**) Same as B, except staining for ELMOD2 and HSP60. Scale bar = 10 μm. (**E**) Mitochondrial ARL2 pixel intensity was quantified in wild type, *mfn1*^*-/-*^, *mfn2*^*-/-*^, or *mfn1*^*-/-*^*mfn2*^*-/-*^ MEFs, as described under Materials and Methods. For each condition,10 cells were analyzed, with the exception of wild type cells, where 11 cells were analyzed. The increases in ARL2 signal in *mfn2*^*-/-*^ and *mfn1*^*-/-*^*mfn2*^*-/-*^ MEFs were statistically significant (p<0.05 and p<0.001, respectively). (**F**) Same as E, except quantified for ELMOD2. Increases in *mfn2*^*-/-*^ and *mfn1*^*-/-*^*mfn2*^*-/-*^ MEFs were statistically significant (p<0.001 and p<0.01, respectively). (**G**) Lysates from wild type, *mfn1*^*-/-*^, *mfn2*^*-/-*^, or *mfn1*^*-/-*^*mfn2*^*-/-*^ MEFs were resolved by SDS-PAGE and transferred to nitrocellulose, as described under Methods. Ponceau S staining (lower panel) was performed immediately after transfer. The membrane was then cut and probed for ARL2 (top panel) or ELMOD2 (middle panel).

**Fig 3:**
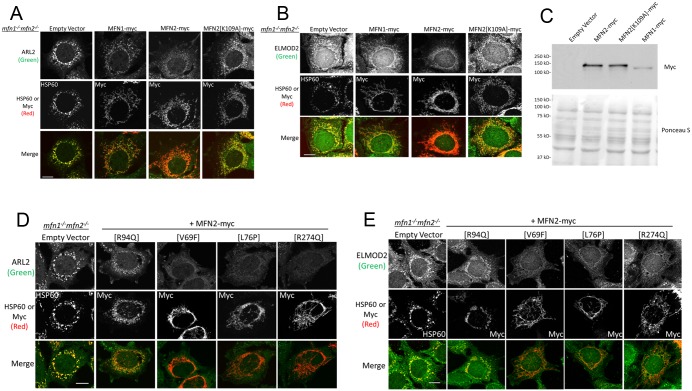
Elevated mitochondrial ARL2 and ELMOD2 is reversed in *mfn1*^*-/-*^*mfn2*^*-/-*^ MEFs with restoration of fusion by MFN2. (**A**) *mfn1*^*-/-*^*mfn2*^*-/-*^ MEFs were transfected with empty vector, or the same vector directing expression of MFN1-myc, MFN2-myc, or MFN2[K109A]-myc. MEFs were fixed 48 hours later and stained for ARL2 (top) and either HSP60 (empty vector) or myc (MFN1-myc, MFN2-myc, MFN2[K109A]-myc) (middle). Merged images are shown at the bottom. Single planes are shown. (**B**) Same as A, except cells were stained for ELMOD2. Z stack projections are shown. (**C**) Cells treated as in A but after 24 hours total cell lysates were obtained, resolved by SDS-PAGE, transferred to nitrocellulose, and probed with anti-myc (upper panel), as described under Methods. Ponceau S staining (lower panel) was performed immediately after transfer. (**D**) *mfn1*^*-/-*^*mfn2*^*-/-*^ MEFs were transfected with empty vector, or the same vector directing expression of MFN2[R94Q]-myc, MFN2[V69F]-myc, MFN2[L76P]-myc, or MFN2[R274Q]-myc. MEFs were fixed 48 hours later and stained for ARL2 (top) and either HSP60 (empty vector) or myc (MFN2-myc) (middle). Merged images are shown (bottom). Single planes are shown. (**E**) Same as D, except cells were stained for ELMOD2. Z stack projections are shown. Scale bars = 10 μm.

**Fig 4:**
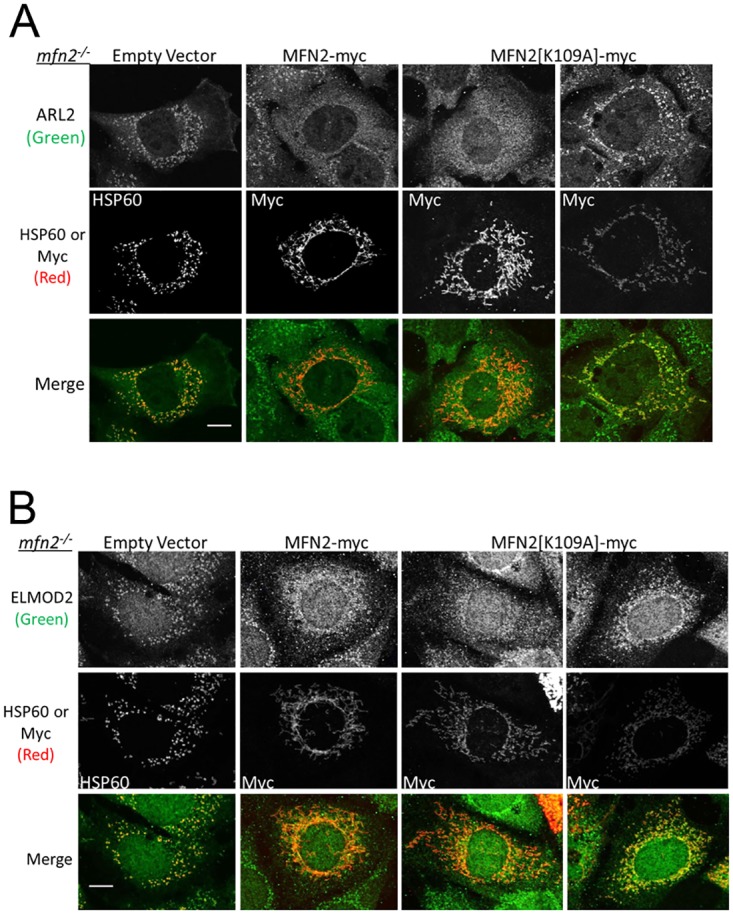
Elevated mitochondrial ARL2 and ELMOD2 are reversed with expression of MFN2-myc or MFN2[K109A]-myc in *mfn2*^*-/-*^ MEFs. (**A**) MFN2-myc or MFN2[K109A]-myc were expressed in *mfn2*^*-/-*^ MEFs, fixed 48 hours later and stained for ARL2 (top) and either HSP60 (empty vector) or myc (MFN2-myc, MFN2[K109A]-myc) (middle). Merged images are shown at the bottom. Single planes are shown. (**B**) Same as A, except cells were stained for ELMOD2. Z stack projections are shown. Scale bar = 10 μm.

**Fig 5:**
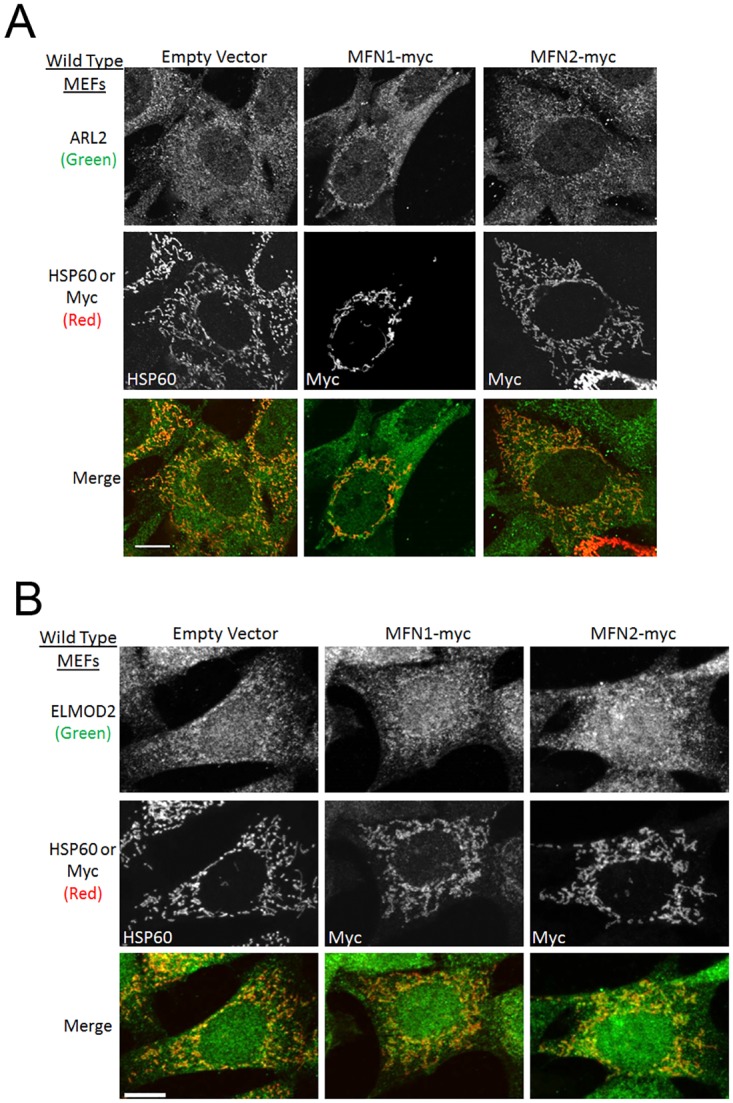
Expression of MFN1-myc or MFN2-myc does not affect ARL2 or ELMOD2 staining intensity in wild type MEFs. (A) Wild type MEFs were transfected with empty vector, MFN1-myc, or MFN2-myc. Cells were fixed 48 hours later and stained for ARL2 (top) and either HSP60 (empty vector) or myc (MFN1-myc, MFN2-myc) (middle). Merged images are shown at the bottom. Single planes are shown. (B) Same as A, except cells were stained for ELMOD2. Z stack projections are shown. Scale bar = 10 μm.

**Fig 6:**
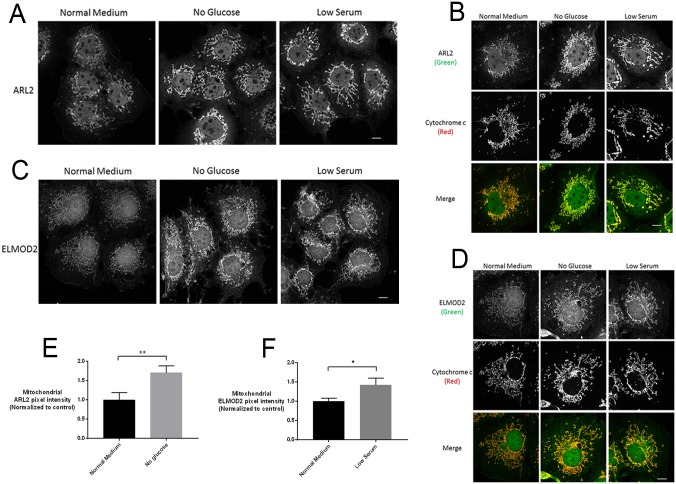
Mitochondrial ARL2 and ELMOD2 increase in cells cultured in 0 glucose or 2% serum. **(A)** COS7 cells cultured for 48 hours in normal, 0 glucose, or 2% serum medium were fixed and stained for ARL2 (top). (**B**) Individual cells stained for ARL2 (top) and cytochrome c (middle) are shown. Merged images are shown at the bottom. Images in A and B are single planes. (**C**) Same as A, except cells were stained for ELMOD2. (**D**) Individual cells stained for ELMOD2 (top) and cytochrome c (middle) are shown, with merged images at the bottom. Images in C and D are z stack projections. **(E)** Mitochondrial ARL2 pixel intensity was quantified in images of cells cultured in normal or no glucose media, as described in Materials and Methods, and the increase was statistically significant (p<0.01). (**F**) Mitochondrial ELMOD2 pixel intensity was quantified in images of cells cultured in normal or low serum media, as described in Materials and Methods, and the increase was statistically significant (p<0.05). All scale bars = 10 μm.

**Fig 7:**
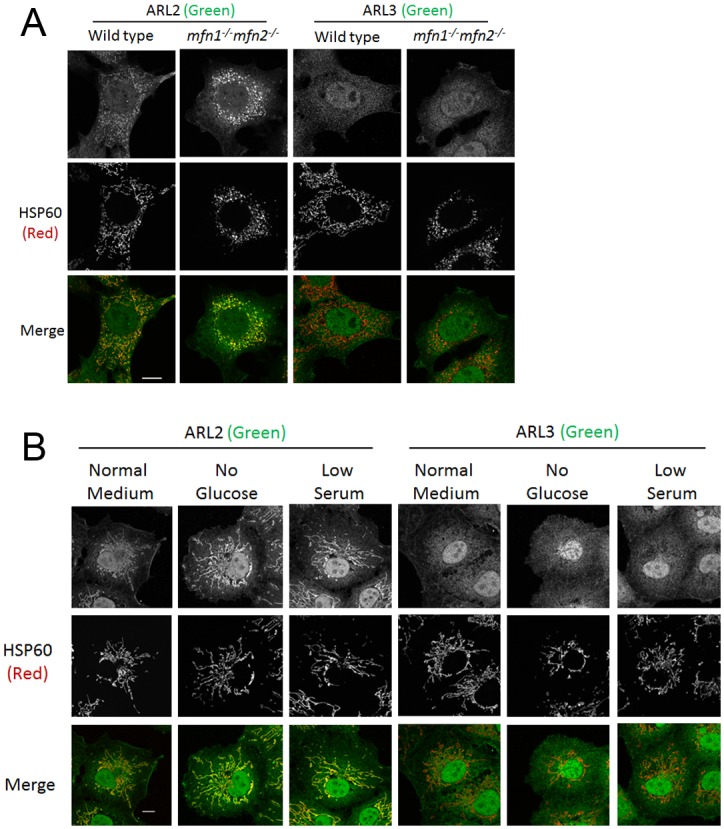
ARL3 staining is unchanged with MFN2 deletion, glucose starvation, or serum starvation. (**A**) Wild type or *mfn1*^*-/-*^*mfn2*^*-/-*^ MEFs were fixed (50%) one day after plating and stained for HSP60 (middle) and either ARL2 (left two columns) or ARL3 (right two columns). Single planes are shown. (**B**) COS7 cells were cultured for 48 hours in normal medium, 0 glucose medium, or 2% serum medium then fixed and stained for HSP60 (middle) and either ARL2 (left three columns) or ARL3 (right three columns). Single planes are shown. All scale bars = 10 μm.

**Fig 8:**
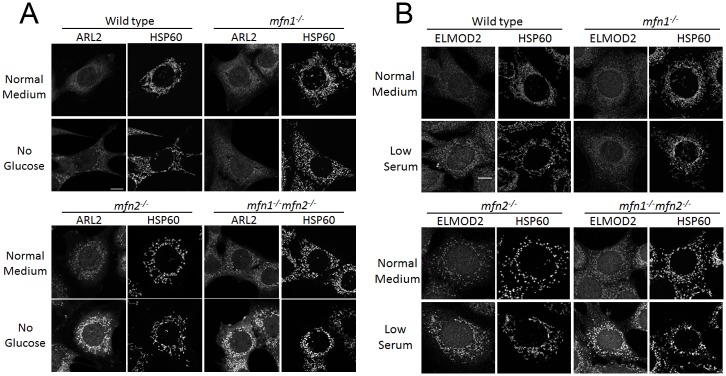
Glucose or serum deprivation increases mitochondrial ARL2 in wild type, *mfn2*^*-/-*^, or *mfn1*^*-/-*^*mfn2*^*-/-*^, but not *mfn1*^*-/-*^ MEFs. MEFs were cultured for 48 hours in normal, 0 glucose, or low serum medium, and fixed and stained for ARL2 (**A**) or ELMOD2 (**B**) as well as HSP60. Single planes are shown in (A) and z stack projections in (B). Scale bar = 10 μm.

**Fig 9:**
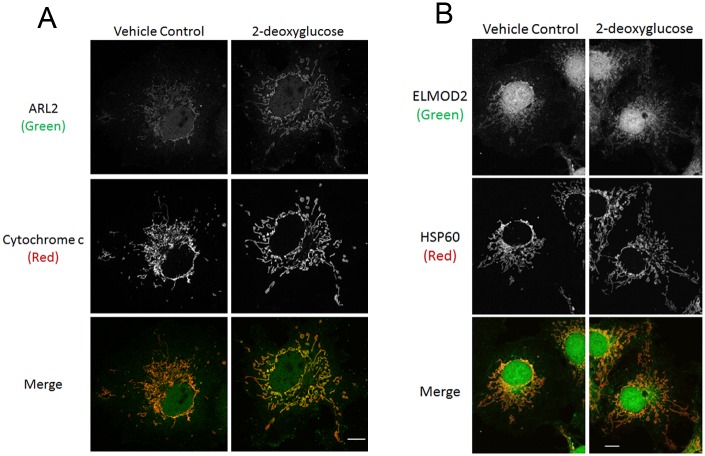
Mitochondrial ARL2, but not ELMOD2, is increased by 2-deoxyglucose. **(A)** COS7 cells were cultured in glucose or 2-deoxyglucose (25 mM) medium. Cells were fixed 16 hours later and stained for ARL2 (top) and cytochrome c (middle). Merged images are shown at the bottom. Images are single planes. **(B)** Same as (A), except cells were stained for ELMOD2. Images are z stack projections. Scale bar = 10 μm.

The first variable identified was the time between plating of cells and fixation prior to imaging. When HeLa cells were fixed and stained within 24 hours of plating, the ARL2 staining profiles were diffuse and only weakly reminiscent of mitochondria, though double labeling with mitochondrial markers confirmed that some ARL2 was indeed overlapping with markers of mitochondria and represented mitochondrial ARL2 (Fig 1A and 1B). Mitochondrial ARL2 staining became stronger and as a result more clearly overlapping with that of the intermembrane space (IMS) marker cytochrome c or matrix marker HSP60 (Fig 1B) after two days and even more so by the third day. This does not appear to be the result of depletion of nutrients from culture medium as more frequent feeding with fresh medium did not alter this time course. By the fourth day after plating or if the initial plating was at a higher cell density (Fig 1B), cells were typically near confluence and we found that mitochondrial staining of ARL2 was clearly decreased. When the time after plating was held constant but the cell density was varied it was quite clear that higher cell density resulted in decreased mitochondrial staining; typically, this was evident as cells approached ~90% confluence. Note that the effects of time after plating and cell density are quite obvious by visual inspection and consistent throughout the cell population, as seen in the view shown in Fig 1A and quantified in Fig 1C, but are more evident as mitochondrial-specific in the enlarged view of single cells, shown in Fig 1B and later figures. The effects of days after plating on ARL2 staining at mitochondria was evident in HeLa and MEFs but not COS7 cells, although the effects of cell density on ARL2 was observed in all three cell lines. Mitochondrial staining of ELMOD2, an ARL2 GAP, also diminished with approach to confluence (Fig 1D) but was unchanged with different days after plating (Fig 1E). When quantified, we observed no statistically significant change in mitochondrial ELMOD2 staining with different days after plating, unlike ARL2. While consistent and highly reproducible, the effects of time after plating and cell density on ARL2 staining were weaker than other effects on ARL2 staining, described below. All immunofluorescence data described used fixation with 4% paraformaldehyde and permeabilization with 0.1% Triton X-100. These conditions were chosen as optimal for visualization of mitochondrial proteins, particularly ARL2 as its larger cytosolic pool is expected to be incompletely fixed and appear as diffuse staining throughout the cell. However, it is also quite apparent that under these conditions our rabbit, polyclonal antibody raised against ELMOD2 yields a strong signal in the nucleus (Figs 1–9). This nuclear staining is present in the pre-immune serum and is not competed by prior incubation of the primary antibody with antigen (purified, recombinant ELMOD2) [25] and thus is non-specific. This is in contrast to the signal from this same antibody that overlaps with mitochondria markers, *e*.*g*., HSP60 (*e*.*g*., Figs 1, 2D, and 3B). All comparisons in staining intensities described below are controlled for time after plating and for cell densities.

### Deletion of MFN2 increases mitochondrial staining of ARL2 and ELMOD2

Using immortalized MEFs deleted for MFN1, MFN2, or both mitofusins [6, 7, 32, 33], we found that expression of the dominant active point mutant, ARL2[Q70L], reversed mitochondrial fragmentation associated with the loss of either MFN1 or MFN2, but not the loss of both (Newman, et al., submitted). When we stained wild type, *mfn1*^*-/-*^, *mfn2*^*-/-*^, or *mfn1*^*-/-*^*mfn2*^*-/-*^ MEFs, all grown under the same conditions (DMEM medium, 25 mM glucose, 10% FBS), we found strong differences in the levels of endogenous, mitochondrial ARL2 staining. MEFs were plated at the same density, and fixed, stained, and imaged in parallel, to control for other factors, as described above. While we observed mitochondrial staining of ARL2 in wild type MEFs, it was clearly stronger in *mfn2*^*-/-*^ MEFs, and even stronger in the double null (*mfn1*^*-/-*^*mfn2*^*-/-*^) MEFs (Fig 2A, 2B and 2E). Though most *mfn1*^*-/-*^*mfn2*^*-/-*^ MEFs had clearly stronger mitochondrial staining of ARL2, compared to *mfn2*^*-/-*^ or wild type MEFs (Fig 2A, 2B and 2E), we noted an increase in heterogeneity in mitochondrial staining of ARL2, compared to the other two MEF lines, possibly due to the previously documented heterogeneity among mitochondria within these cells due to the complete loss of fusion [7]. Because of this greater heterogeneity, we show both a field of cells as well as an enlarged view of 1–2 cells in Fig 2A and 2B, respectively, with the latter showing double labeling with the matrix marker HSP60. We obtained the same results when ARL2 was visualized with a mouse monoclonal antibody, detecting the increases in staining of mitochondrial ARL2 in *mfn2*^*-/-*^ and *mfn1*^*-/-*^*mfn2*^*-/-*^ MEFs. In striking contrast, mitochondrial ARL2 staining in *mfn1*^*-/-*^ MEFs appeared diminished, compared to wild type MEFs, with the majority of cells in the population showing no discernable mitochondrial ARL2 signal (Fig 2A and 2B). Quantification of wild type and *mfn1*^*-/-*^ MEFs revealed no significant differences between these two populations, while the *mfn2*^*-/-*^ and double null cells displayed statistically significantly increases in ARL2 staining than wild type controls (Fig 2E). Fig 2 also shows, particularly when viewing HSP60 staining, the fragmentation of mitochondria that results from the loss of MFNs, as described previously [6, 7]. Because mitochondrial staining was increased in *mfn2*^*-/-*^ and *mfn1*^*-/-*^*mfn2*^*-/*^ MEFs, but unchanged in *mfn1*^*-/-*^ MEFs, the increase in mitochondrial signal was clearly not simply an indirect result of mitochondrial fragmentation, but was linked to MFN2.

ELMOD2 was first identified as an ARL2 GTPase activating protein (GAP) [26] and later evidence pointed to it acting as an ARL2 effector in mitochondria [25]. Thus, we asked if mitochondrial ELMOD2 staining is also sensitive to MFN2 deletion, and found that it too is elevated in MEFs deleted for MFN2 over that seen in wild type cells (Fig 2C, 2D and 2F). The double null *mfn1*^*-/-*^*mfn2*^*-/-*^ cells also displayed higher staining than seen in the wild type MEFs, though this was not clearly or consistently higher than that seen in *mfn2*^*-/-*^ cells (Fig 2C, 2D and 2F). The ELMOD2 signal was not obviously weaker in the *mfn1*^*-/-*^ cells than in wild type, perhaps due to the overall weak mitochondrial staining of ELMOD2 in wild type MEFs (Fig 2C, 2D and 2F). Thus, ARL2 and ELMOD2 staining in mitochondria are each specifically increased in cells deleted for MFN2. We observed no evident changes in HSP60 (Fig 2B and 2D) or cytochrome c signals between these MEF lines, supporting the conclusion that the changes in staining were specific to ARL2 and ELMOD2. Immunoblots of total cell lysates from wild type, *mfn1*^*-/-*^, *mfn2*^*-/-*^, or *mfn1*^*-/-*^*mfn2*^*-/-*^ MEFs showed no differences in the levels of endogenous ARL2 or ELMOD2 between these different cell lines (Fig 2G), supporting our conclusion that the mitochondrial pools of ARL2 and ELMOD2 were specifically altered. The effects of gene deletions on ARL2 and ELMOD2 at mitochondria are summarized in Table 1.

**Table 1:** Summary of effects of MFN deletions on ARL2/ELMOD2 staining in MEF lines.

MEF line	Transfected plasmid (Protein expressed)	ARL2 staining relative to wild type MEFs	ELMOD2 staining relative to wild type MEFs
Wild type	N/A	NC	NC
*mfn1*^*-/-*^	N/A	NC	NC
*mfn2*^*-/-*^	N/A	+	+
*mfn1*^*-/-*^*mfn2*^*-/-*^	N/A	+	+
*mfn2*^*-/-*^	Empty vector	+	+
*mfn2*^*-/-*^	MFN1-myc	+	+
*mfn2*^*-/-*^	MFN2-myc	-	-
*mfn2*^*-/-*^	MFN2[K109A]-myc	-	-
*mfn1*^*-/-*^*mfn2*^*-/-*^	Empty vector	+	+
*mfn1*^*-/-*^*mfn2*^*-/-*^	MFN1-myc	+	+
*mfn1*^*-/-*^*mfn2*^*-/-*^	MFN2-myc	-	-
*mfn1*^*-/-*^*mfn2*^*-/-*^	MFN2[K109A]-myc	+	+
*mfn1*^*-/-*^*mfn2*^*-/-*^	MFN2[R94Q]-myc	+	+
*mfn1*^*-/-*^*mfn2*^*-/-*^	MFN2[V69F]-myc	-	-
*mfn1*^*-/-*^*mfn2*^*-/-*^	MFN2[L76P]-myc	-	-
*mfn1*^*-/-*^*mfn2*^*-/-*^	MFN2[R274Q]-myc	-	-

See text for details. NC, no change compared to wild type MEFs. +, - indicate clearly increased or decreased mitochondrial staining, respectively.

### Increased mitochondrial staining of ARL2 and ELMOD2 in mfn1^-/-^mfn2^-/-^ MEFs is reversed upon expression of MFN2 and restoration of tubular morphology

As a further test of specificity of the effects of MFN2 deletion, we performed rescue experiments. Transfection of *mfn1*^*-/-*^*mfn2*^*-/-*^ MEFs with pcDNA3.1 (empty vector) resulted in no changes in either mitochondrial morphology or ARL2 staining (Fig 3A, left). Expression of MFN2-myc in these cells restored tubular mitochondrial morphology, as previously reported [34]. MFN2-myc also reversed the increased ARL2 staining of mitochondria (Fig 3A). The differences between the mitochondrial staining of ARL2 in *mfn1*^*-/-*^*mfn2*^*-/-*^ cells expressing MFN2-myc, compared to empty vector control, is perhaps the strongest change in staining of ARL2 that we observed in this study. Because transient transfection yields a mixed population in terms of transfected cells and in levels of protein expression, it was quite apparent that there was a strong correlation between the ability of MFN2-myc expression to reverse mitochondrial fragmentation and elevated mitochondrial staining of ARL2. In most cells with tubular morphology, ARL2 staining was reduced to the point where it was barely detectible and lower even than ARL2 staining in wild type MEFs (Fig 2A). As mitochondrial staining of ARL2 is reduced, the cytosolic staining becomes more evident (e.g., see Fig 3A, top MFN2-myc panel), thus the loss in mitochondrial ARL2 signal is perhaps best seen in the merged image, revealing the clear loss in overlap with myc staining, which is marking MFN2 and mitochondria. Though a minority of MFN2-myc expressing cells with tubular mitochondrial morphology still displayed mitochondrial staining of ARL2, this staining was clearly diminished compared to controls. In MFN2-myc positive cells with fragmented mitochondria, ARL2 staining remained high, suggesting that the effect was correlated with reversal of fragmentation.

We next investigated effects of MFN2 expression on mitochondrial ELMOD2, and found that MFN2-myc expression could also reverse the elevated ELMOD2 (Fig 3B). MEFs deleted for both MFNs displayed strong ELMOD2 staining that overlapped extensively with that of HSP60 (Fig 3B, left panels). In contrast, the double MFN null cells expressing MFN2-myc had clearly reduced staining of mitochondrial ELMOD2, as evidenced by the lack of overlap between the ELMOD2 and myc staining (Fig 3B). And as was true for ARL2, we observed a strong correlation between rescue of fragmentation by MFN2 and the loss of mitochondrial staining of ELMOD2. Similarly, the small percentage of *mfn1*^*-/-*^*mfn2*^*-/-*^ MEFs that expressed low levels of MFN2-myc and retained fragmented mitochondria displayed high levels of mitochondrial ELMOD2 staining that was comparable to cells receiving empty vector. Similar results were obtained when cells were examined 24 or 48 hours after transfection.

Because the reversal of elevated mitochondrial ARL2/ELMOD2 staining in *mfn1*^*-/-*^*mfn2*^*-/-*^ MEFs by MFN2-myc was correlated with restoration of tubular morphology, we asked if the reversal depended on the fusogenic activity of the expressed MFN2. A mutation in the GTPase domain of MFN2, [K109A], abolishes its fusogenic activity. We confirmed the failure of MFN2[K109A]-myc to reverse the fragmentation phenotype in *mfn1*^*-/-*^*mfn2*^*-/-*^ MEFs [34] (Fig 3). No changes in the staining of ARL2 or ELMOD2 were observed in cells expressing MFN2[K109A]-myc, compared to empty vector controls. ARL2 and ELMOD2 remained mitochondrial, indistinguishable in intensity from controls, and co-localized with myc staining (Fig 3). Results were the same when cells were fixed 24 or 48 hours after transfection. Therefore, we consistently observed close correlations between staining intensities of ARL2 and ELMOD2 in mitochondria and fusion activity of MFN2; *e*.*g*., the ability of MFN2 to reverse the increased ARL2/ELMOD2 mitochondrial signal requires its fusogenic activity.

Expression of MFN1-myc also reversed fragmentation in *mfn1*^*-/-*^*mfn2*^*-/-*^ MEFs, as previously reported [34]. But in marked contrast to MFN2-myc, MFN1-myc did not reverse the increased staining of mitochondrial ARL2 (Fig 3A) or ELMOD2 (Fig 3B). Thus, the reversal of elevated ARL2 staining seen with MFN2-myc expression was not due simply to restoration of tubular morphology but was specific to, and perhaps dependent upon, MFN2-mediated fusion.

To ensure that the changes in mitochondrial ARL2 and ELMOD2 were not simply due to differences in expression between MFN2-myc, MFN2[K109A]-myc, and MFN1-myc, we performed immunoblots, probing for the myc epitope after expression in *mfn1*^*-/-*^*mfn2*^*-/-*^ MEFs. Immunoblots showed no differences in the levels of expression between MFN2-myc and MFN2[K109A]-myc (Fig 3C). The signal detected for MFN1-myc was lower than that seen for MFN2-myc, but the MFN1 fusion protein contains 10 myc tags on its C terminus, while each of the MFN2 proteins carries 16 myc tags. The increase in number of epitope tags is expected to increase the sensitivity in immunoblots using the myc antibody and also likely explains the slightly faster mobility of MFN1-myc in these gels (Fig 3C). Thus, though we are unable to definitively conclude that MFN1-myc and MFN2-myc express to similar levels, it is likely that they are expressed similarly as the same vector was used and they each rescue the mitochondrial morphology defects observed in the deletion strains.

Mutations in MFN2 cause Charcot Marie Tooth Type 2A, a peripheral neuropathy [35]. Though most disease-associated mutations (such as [R94Q]) interfere with the fusogenic activity of MFN2, a subset (e.g., [V69F], [L76P], [R274Q]) are still capable of fusion [34]. We asked if these MFN2 mutants, which are still capable of fusion, have differential effects on ARL2 or ELMOD2 staining at mitochondria, compared to MFN2-myc. We expressed myc-tagged MFN2 harboring [V69F], [L76P] or [R274Q] mutations in *mfn1*^*-/-*^*mfn2*^*-/-*^ MEFs and found that each MFN2 mutant restored tubular morphology, as previously described [34]. Furthermore, each mutant also reversed the increased ARL2 (Fig 3D) or ELMOD2 (Fig 3E) staining at mitochondria, similar to wild type MFN2-myc, at both 24 and 48 hours after transfection. In contrast, MFN2[R94Q]-myc failed to rescue fragmentation, consistent with previously published data [34], and did not reverse the elevated ARL2 and ELMOD2 staining in *mfn1*^*-/-*^*mfn2*^*-/-*^ MEFs (Fig 3E and 3F). We conclude that changes in ARL2 and ELMOD2 staining at mitochondria are closely linked to MFN2-mediated fusion.

### Reversal of elevated ARL2 and ELMOD2 staining in mfn2^-/-^ MEFs

Expression of MFN2-myc in *mfn2*^*-/-*^ MEFs led to restoration of tubular mitochondria, as well as a decrease in mitochondrial ARL2/ELMOD2 (Fig 4A and 4B), similar to our results in *mfn1*^*-/-*^*mfn2*^*-/-*^ MEFs. In contrast to results in the double null cells, expression of MFN2[K109A]-myc in *mfn2*^*-/-*^ MEFs restored tubular mitochondrial morphology in ~50% of transfected cells, consistent with previously reported data [6]. The partial rescue by MFN2[K109A]-myc in *mfn2*^*-/-*^ MEFs, compared to the lack of any rescue of fragmentation in *mfn1*^*-/-*^*mfn2*^*-/-*^ MEFs, is likely due to the presence of endogenous MFN1 in *mfn2*^*-/-*^ MEFs, as MFN1 and MFN2 can hetero-oligomerize to promote fusion [6, 7, 36]. In MEFs expressing MFN2[K109A]-myc and displaying tubular morphology, we observed a decrease in ARL2 and ELMOD2 mitochondrial signal, comparable to expression of MFN2-myc (Fig 4A and 4B). However, in the remaining cells expressing MFN2[K109A]-myc that retained fragmented mitochondria, mitochondrial staining of ARL2/ELMOD2 remained high (Fig 4A and 4B right most panels). Cells expressing MFN2[K109A]-myc with fragmented mitochondria generally appeared to have lower levels of myc expression (Fig 4A and 4B), possibly explaining the lack of rescue in those cells. We conclude that the increase in mitochondrial staining of ARL2 and ELMOD2 that is so evident in cells deleted for MFN2, alone or in combination with MFN1, is dependent on the ability of MFN2 to support fusion in MEFs.

### Expression of MFN2-myc in wild type MEFs does not alter mitochondrial staining of ARL2 or ELMOD2

As re-introduction of MFN2 in *mfn1*^*-/-*^*mfn2*^*-/-*^ MEFs caused a decrease in mitochondrial staining of ARL2, we tested if MFN2-myc could also decrease endogenous mitochondrial ARL2 or ELMOD2 in wild type MEFs. Empty vector, MFN1-myc, or MFN2-myc were expressed in wild type MEFs, which were fixed and stained for HSP60 and ARL2 or ELMOD2 24 or 48 hours later. MEFs receiving empty vector displayed tubular mitochondrial morphology, whereas MFN1-myc and MFN2-myc led to elongation of mitochondria. Neither empty vector, MFN1-myc, nor MFN2-myc had any obvious effect on mitochondrial staining of ARL2 or ELMOD2 in wild type cells, which remained weak (Fig 5). Thus, there may exist minimal levels of mitochondrial ARL2 and ELMOD2 which are not subject to regulation by the levels of MFN2.

### Mitochondrial ARL2 and ELMOD2 increase in response to stressors that cause mitochondrial elongation

Mitochondrial elongation occurs in response to stressors, such as glucose deprivation, as a result of increased fusion and decreased fission [10, 12, 37, 38]. We asked if mitochondrial staining of ARL2 or ELMOD2 changed in response to such conditions, and if these effects were correlated with changes to mitochondrial morphology. We found that ARL2 in mitochondria was markedly increased under a number of conditions, summarized in Table 2. Two of the conditions that yielded among the most striking changes were growth of COS7 cells in the absence of glucose (0 glucose), or in low (2%) serum (Fig 6A, 6B and 6D). Our cells are routinely maintained in DMEM that contains 25 mM glucose supplemented with 10% fetal bovine serum (FBS). Effects were evident one day after changing the medium to 0 glucose or 2% FBS and were further increased at two days. In contrast to the marked differences in ARL2 staining, no changes in intensity were observed with several other mitochondrial markers, including HSP60 (matrix), cytochrome c (IMS), TOM20 (outer membrane), Complex I subunit NDUFA9 (inner membrane), Complex III subunit UQCRC2, and Complex V subunit alpha. Similar, though somewhat less striking effects on ARL2 staining were observed when cells were cultured for 48 hours in 10 mM galactose with 0 glucose, which makes cells more dependent upon respiration instead of glycolysis [39], with no changes to mitochondrial markers.

**Table 2:** Summary of the effects of energetic stressors on mitochondrial staining of ARL2 or ELMOD2.

Stressor	Duration of stress	ARL2 staining	ELMOD2 staining	Effect on mitochondrial morphology	Cell lines
0 mM glucose + 10% FBS	24, 48 hours	+	+	Elongation	COS7, HeLa, MEFs
25 mM glucose + 2% FBS	24, 48 hours	+	+	Elongation	COS7, HeLa, MEFs
10 mM galactose	24, 48 hours	+	+	Elongation	COS7, HeLa, MEFs
25 mM 2-deoxyglucose	16 hours	+	NC	Partial fragmentation	COS7, HeLa, MEFs
10 μM oligomycin	16 hours	+	NC	Fragmentation	COS7, HeLa, MEFs
10 μM antimycin A	16 hours	+	NC	Fragmentation	COS7, HeLa, MEFs
10 μM rotenone	16 hours	+	NC	Fragmentation	COS7, HeLa, MEFs
10 μM menadione	1 hour	+	NC	No change	COS7, HeLa

See text for details. + indicates an increase in staining intensity, over that seen in cells grown in DMEM medium with 25 mM glucose and 10% FBS. NC indicates no changes in staining intensities were evident.

Mitochondrial staining of ELMOD2 was also increased when cells were grown in 0 glucose or 2% serum (Fig 6C, 6D, and 6F). The kinetics of these effects were similar to those seen for ARL2, with ELMOD2 staining increased after 24 hours, and more so after 48 hours in all three cell lines. Also like ARL2, when cells were grown in galactose containing medium (10 mM, with 0 glucose), ELMOD2 staining was increased within 24 hours and further increased after 48 hours. The increase in ELMOD2 staining seen in galactose was less prominent than the effect with 0 glucose or low serum, again similar to ARL2.

In addition to the increased staining of ARL2 and ELMOD2 under the conditions described above, we also observed changes in mitochondrial morphology resulting from the treatments used. Growth in 0 glucose, 2% serum, or 10 mM galactose each resulted in an increase in mitochondrial elongation, branching, and looping structures, compared to controls (Fig 6). This effect was most obvious with 0 glucose or in galactose medium, and was observed in COS7, HeLa, and MEFs, though was most obvious in MEFs, due to mitochondria appearing more elongated in untreated COS7 and HeLa cells. These changes in mitochondrial morphology are consistent with previously published data in regards to cellular starvation [9–12, 38, 40]. We also note that mitochondrial staining of endogenous ARL2 and ELMOD2 appeared stronger in COS7 cells (Fig 6) than in HeLa (Fig 1) or MEFs (Fig 2). This may be due to different mitochondrial morphologies across the three cell lines.

### ARL3 staining does not change with MFN2 deletion or cellular stressors

As ARL2 is a member of the ARF family of GTPases, we wondered if the effects of MFN2 deletion were unique to ARL2, or if MFN2 deletion could also recruit other ARF family GTPases to mitochondria. ARL3 is the closest paralog to ARL2 in humans, sharing 53% identity with ARL2. Therefore, we stained wild type or *mfn1*^*-/-*^*mfn2*^*-/-*^ MEFs, where we had seen the largest increase in ARL2 staining, for ARL3 and did not observe any mitochondrial localization of ARL3 (Fig 7A), consistent with previously reported data [25]. We performed the same experiment staining cells for ARL13B, or ARF1-6, and saw no mitochondrial localization of any other GTPase in either wild type or *mfn1*^*-/-*^*mfn2*^*-/-*^ MEFs. As a further test of specificity, we cultured COS7 cells in 0 glucose or low serum media, and stained cells for ARL3. While each of these conditions led to an increase in mitochondrial ARL2, no changes were observed in ARL3 staining with either condition (Fig 7B). Therefore, mitochondrial localization of ARL2 is unique among ARF family GTPases, as are the effects of MFN2 deletion and stressors on mitochondrial ARL2.

### ARL2 and ELMOD2 are further increased in stressed mfn2^-/-^ and mfn1^-/-^mfn2^-/-^ MEFs, but not mfn1^-/-^ MEFs

Given that deletion of MFN2 or starving cells each increase ARL2 staining in mitochondria, we asked if these effects were additive. We grew wild type, *mfn1*^*-/-*^, *mfn2*^*-/-*^, or *mfn1*^*-/-*^*mfn2*^*-/-*^ MEFs in normal, 0 glucose, or low serum media for two days before fixing and processing. The wild type MEFs grown in either 0 glucose or low serum had increased ARL2 and ELMOD2 mitochondrial signal compared to those grown in normal medium (Fig 8), and displayed elongated mitochondria, as described above in COS7 and HeLa cells. MEFs deleted for MFN2 cultured in 0 glucose or 2% serum had increased ARL2 or ELMOD2 mitochondrial staining compared to normal medium (Fig 8). Similarly, *mfn1*^*-/-*^*mfn2*^*-/-*^ MEFs grown in 0 glucose or low serum media also had stronger ARL2 (Fig 8A) and ELMOD2 (Fig 8B) mitochondrial staining over that seen in normal medium. For each of the MEF lines, 0 glucose resulted in the strongest increase in ARL2 staining, while growth in 2% serum resulted in the strongest increase in ELMOD2 staining. In contrast, there was little or no increase in ARL2 or ELMOD2 in *mfn1*^*-/-*^ MEFs grown in no glucose or low serum medium for 48 hours, compared to *mfn1*^*-/-*^ MEFs grown in normal medium (Fig 8).

We also observed striking differences in mitochondrial morphology in response to stress between the MEF lines. MFN2 null MEFs grown in normal medium had fragmented mitochondria, compared to wild type cells, but growth in 0 glucose or low serum resulted in a partial reversal in mitochondrial morphology, displaying an intermediate, rod-shaped morphology, with some cells displaying a tubular morphology (Fig 8). In contrast, *mfn1*^*-/-*^ MEFs showed no evidence of even partial rescue of the fragmentation resulting from the absence of MFN1 when grown in 0 glucose or low serum. Finally, *mfn1*^*-/-*^*mfn2*^*-/-*^ MEFs cultured in 0 glucose or low serum also displayed no differences in mitochondrial morphology compared to cells grown in normal medium, consistent with their inability to undergo fusion [7].

### Metabolic inhibitors increase mitochondrial ARL2 but not ELMOD2

Mitochondrial fusion is linked to the activity of the electron transport chain [10]. Therefore, we tested other metabolic stressors for effects on mitochondrial ARL2 levels. Every metabolic poison acting on ATP generation that we tested resulted in increased mitochondrial ARL2. The strongest effect was observed after an overnight (16 hr) treatment with 2-deoxyglucose (25 mM), an inhibitor of glycolysis [41] (Fig 9A). Similar effects were seen when cells were treated with specific inhibitors of the electron transport chain; oligomycin (10 μM, complex V inhibitor), rotenone (10 μM, complex I inhibitor), or antimycin A (10 μM, complex III inhibitor). Time courses were performed to determine the maximal effects of 2-deoxyglucose, oligomycin, or antimycin A. We found that increased ARL2 was evident as soon as 3 hours after addition of each drug and continued to increase, peaking at 16 hours. Cells treated with rotenone did not show any obvious increase until overnight (16 hr) treatment. We also observed an increase in ARL2 staining with 1 hour of menadione treatment, which increases mitochondrial ROS [42]. These effects were observed in all three cell lines examined—COS7, HeLa, and MEFs (Table 2).

In marked contrast to ARL2, we found that ELMOD2 staining was not altered in response to the same drugs that caused mitochondrial ARL2 to increase. Overnight treatment of cells with 2-deoxyglucose (Fig 9B), oligomycin, antimycin A, rotenone, or menadione had no effect on ELMOD2 staining (Table 2). None of these drugs caused mitochondrial elongation. Oligomycin, antimycin A, and rotenone caused mitochondrial fragmentation, while 2-deoxyglucose and menadione had minimal effects on mitochondrial morphology. Therefore, mitochondrial staining of ARL2 responds to metabolic stress, but staining of ELMOD2 only changes when mitochondrial elongation is observed.

### Mitochondrial ARL2 is not altered by stressors when the recombinant protein is directed to mitochondria

**Fig 10:**
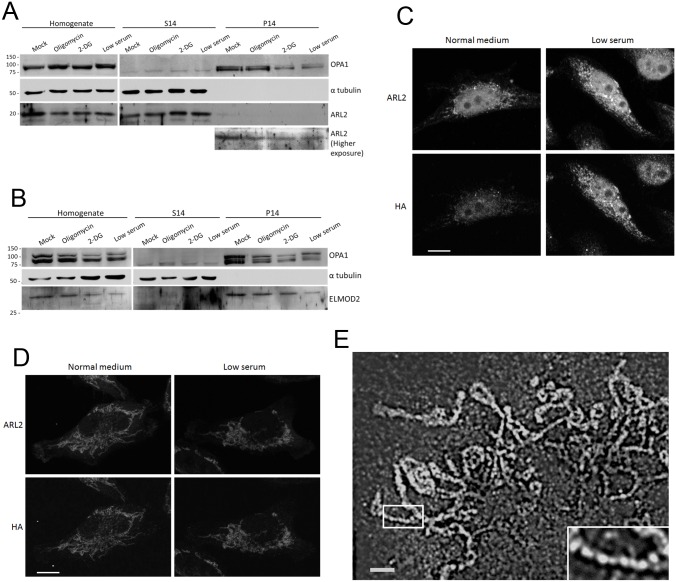
ARL2 and HA staining increase in stressed cells expressing ARL2-HA, but not in cells expressing SMAC-HA-ARL2. **(A)** HeLa cells were treated overnight with 25 mM 2-deoxyglucose, 10 μM oligomycin or vehicle control, or grown in medium with 2% serum for 48 hours. Cells were then harvested and fractionated as described under Materials and Methods. Homogenate, S14, and P14 fractions were immunoblotted for ARL2, alpha tubulin (cytosolic marker), and OPA1 (mitochondrial marker). Equal volumes were loaded. **(B)** Same as A except fractions were immunoblotted for ELMOD2.(**C**) HeLa cells were transfected with ARL2-HA and allowed to attach to coverslips for 4 hours, before the medium was replaced with normal or 2% serum medium. Two days later, cells were permeabilized prior to fixation, to remove cytosolic ARL2, then stained for ARL2 and HA. Images are z stack projections. **(D)** HeLa cells expressing SMAC-HA-ARL2 were treated as in A but without pre-fixation permeabilization. Cells were fixed and stained for ARL2 and HA. Images are single planes acquired by confocal microscopy. Scale bar = 10 μm. **(E)** COS7 cells were treated with 25 mM 2-deoxyglucose for 16 hr, fixed and stained for ARL2 and imaged using SIM. A representative cell is shown. The blowup (bottom right) is 2.5x the size of the larger image shown. Scale bar = 2 μm.

Western blots of whole cell lysates, comparing controls to the same cells after several of the treatments described above, failed to detect any consistent changes in the total cellular ARL2 or ELMOD2 expressed in any of the three cell lines tested; as an example data from HeLa cells are shown in Fig 10A and 10B. When mitochondria were enriched by differential centrifugation, pelleting mitochondria at 14,000x*g* from a post-nuclear supernatant, we also could detect no consistent changes in mitochondria-associated ARL2 or ELMOD2. HeLa cells treated with 2-deoxyglucose or oligomycin or grown in low serum medium showed no increase in ARL2 (Fig 10A) or ELMOD2 (Fig 10B) in the P14. In the immunoblots shown in Fig 10A and 10B, the ARL2 and ELMOD2 immunoreactivity does appear slightly reduced in some conditions, but this is not a consistent finding and in the data shown it is under conditions in which ARL2 and ELMOD2 staining at mitochondria is *increased*. Thus, any increases in immunoreactivity observed at mitochondria by immunofluorescence is not the result of an increase in total cellular ARL2/ELMOD2 or that in the P14. Note that because only a minor fraction of total cellular ARL2 fractionates with mitochondria (P14, Fig 10A) and that ELMOD2 is expressed to only low levels, long exposures are required to monitor their levels by immunoreactivity, which results in higher backgrounds. Similarly, no differences in ARL2 or ELMOD2 were observed between the crude mitochondria fractions from wild type, *mfn1*^*-/-*^, *mfn2*^*-/-*^, or *mfn1*^*-/-*^*mfn2*^*-/-*^ MEFs.

That immunoblots of the crude mitochondria fraction from cells that display increased staining of ARL2/ELMOD2 did not show correlated increases in the protein fractionating with mitochondria is difficult to interpret for a number of reasons. Our initial finding that simply detaching cells from the culture dish, the first step in both re-plating cells and for cell fractionation, may be sufficient to reverse any increases in staining at mitochondria (Fig 1) resulting from the treatments used, suggests that a reversible process is involved. As a result, rapid degradation or export of ARL2/ELMOD2 from mitochondria in response to cell detachment may confound fractionation results. While we believe that the increased import of ARL2 into mitochondria explains the increased ARL2 staining, there exist other possibilities that we sought to test. For example, a time-dependent incorporation of ARL2/ELMOD2 into a macromolecular complex or change in tertiary structure, resulting in altered access of antibodies to specific epitopes upon fixation, could yield the changes in staining intensities identified above.

To ask if changes in antibody recognition of the epitope were involved, we expressed ARL2 carrying a small epitope tag at its C-terminus, ARL2-HA, in HeLa cells and examined changes in HA staining in cells in response to stressors. The C-termini of ARF family proteins are not involved directly in protein-protein interactions and thus the access of antibodies to the HA epitope at that location is predicted to be unaltered by ARL2 binding to partners. Like untagged ARL2, ARL2-HA was partially imported into mitochondria, and brief permeabilization of cells prior to fixation (0.5% saponin, 1 minute) removed cytosolic ARL2 and allowed for better visualization of the mitochondrial pool (Newman, et al., submitted). Note that pre-fixation permeabilization results in changes in mitochondrial morphologies, as they appear smaller and more fragmented than when fixed prior to permeabilization. In addition, cells were imaged using higher laser power, due to loss of signal, so Z stack projections are shown as they average the increase in noise to yield the optimal signal to noise. ARL2-HA was expressed in cells grown in low serum. After two days, cells were permeabilized prior to fixation, and stained for ARL2 and HA. As expected, the low serum treatment increased endogenous mitochondrial ARL2 staining in cells receiving empty vector and also increased mitochondrial HA and ARL2 staining in cells expressing ARL2-HA (Fig 10C). We were unable to perform a similar experiment for ELMOD2 as we found that epitope tagging at either the N or C terminus interfered with its localization to mitochondria. These results are consistent with the low serum treatment causing an increase in the abundance of ARL2-HA at mitochondria and suggest that increased ARL2 staining at mitochondria reflects increased mitochondrial import or half-life of the imported ARL2 rather than a change in antibody access to the fixed ARL2.

We next asked if ARL2 that is strongly driven to the IMS, as a result of an N-terminal fusion with the leader sequence from SMAC (SMAC-HA-ARL2) [43] is affected by stressors. This construct is appropriately and efficiently targeted to the IMS, with rapid cleavage of the SMAC leader and retention of the HA-ARL2 in the IMS with no effects on mitochondrial morphology [43]. SMAC-HA-ARL2 was expressed in HeLa cells, which were replated onto coverslips and allowed to attach for four hours before changing to low serum medium. Two days later, cells were fixed and stained for ARL2 and HA. When ARL2 was imaged in cells receiving empty vector, we saw the expected increase in endogenous ARL2 staining that co-localizes with mitochondrial markers, in response to growth of cells in low serum medium (data not shown). In contrast, in cells expressing SMAC-HA-ARL2, there was already a strong signal for HA or ARL2 from mitochondria and this was not further increased in response to growth in low serum (Fig 10D). Similar results were obtained with OCT-HA-ARL2, which targets ARL2 to the matrix. Taken together, these results suggest that the increased staining of ARL2 at mitochondria reflects increased levels of the protein, resulting from either increased import or decreased loss (degradation or export), rather than changes in tertiary or quaternary structure that might alter immunostaining.

Finally, mitochondrial ARL2 is found in discrete puncta along mitochondria, optimally visualized using structured illumination microscopy (SIM) (Newman et al., submitted). SIM is useful for increasing resolution of fluorescence images but less so for comparing intensities between images due to the requirements for data acquisition and processing. We asked if conditions leading to increased ARL2 in mitochondria were associated with changes in ARL2 puncta. COS7 cells were treated with 2-deoxyglucose overnight, stained for ARL2, and imaged by SIM. We observed that ARL2 still localized to puncta in 2-deoxyglucose treated cells (Fig 10E) repeating along the length of mitochondria at an interval of ~0.4 μm, as previously described for untreated cells (Newman et al., submitted). Therefore, increased mitochondrial ARL2 appears to represent increased abundance of ARL2 at puncta, though we cannot eliminate the possibility of increased amounts of soluble ARL2 inside mitochondria as well.

## Discussion

ARL2 plays essential cellular roles both at mitochondria, where it regulates energy metabolism and fusion [25, 30](Newman et al., submitted), and in cytosol, where together with TBCD it regulates tubulin biogenesis, microtubules, and has links to cell division [44–47]. Because only a small fraction of total cellular ARL2 is found at mitochondria and the majority is tightly bound to TBCD in cytosol there is predicted to be a mechanism for regulating recruitment to mitochondria, though such a mechanism remains elusive. We describe increases in mitochondrial pools of ARL2 in response to a number of environmental factors; including changes in fusogenic activity resulting from deletion of mitofusins (particularly MFN2), cell attachment, cell density, and pharmacological stressors that target energy metabolism. Many, but not all, of the changes observed in mitochondrial pools of ARL2 were also seen for the ARL2 GAP, ELMOD2, though not for any other mitochondrial proteins monitored. Once we were able to control for effects of time after cell attachment and cell density, the changes in ARL2 and ELMOD2 intensities became very clear and consistent in each of the three cell lines used in our studies (see Tables 1 and 2 for summaries). We discuss the interpretation of these results within the context of the existing literature and recent data from our lab demonstrating a role for ARL2 in the regulation of mitochondrial fusion from the IMS (Newman et al., submitted).

What is responsible for the increases in ARL2 and ELMOD2 at mitochondria described here? Previously, we showed that mitochondrial ARL2 is increased in abundance several fold, though only in the affected tissues (heart and skeletal muscle), in *ant1*^*-/-*^ mice [30], which display muscle wasting and cardiomyopathy [48]. This observation was interpreted as evidence supporting the existence of a regulated mitochondrial import pathway and led us to study changes to ARL2 in response to stressors in cell culture. Our more recent studies of the effects of dominant mutants of ARL2 on mitochondrial morphology and motility, upstream of mitofusins (Newman et al., submitted), also led us to investigate effects of changes in MFN levels on mitochondrial ARL2 and ELMOD2. While we see quite strong and uniform increases in ARL2 staining at mitochondria, no changes in ARL2 or ELMOD2 were detected in crude mitochondrial fractions by immunoblotting. However, our observation that simply trypsinizing and replating cells dramatically diminished ARL2 staining at mitochondria confounds interpretation of these results. We considered changes in availability of epitopes as an alternative explanation for the changes in fluorescence intensity. To control for this, we tested whether stress induced increases were evident in cells expressing C-terminal HA tagged ARL2 and found that they were. Because HA imaging used an unrelated antibody and is directed to a tag that is not present in endogenous ARL2, and thus not likely to be directly involved in protein-protein interactions, we interpret this as strongly supporting our conclusion that the increased immunofluorescence signal is the result of increases in the amount of protein at mitochondria. This conclusion was further supported by the observation that another recombinant protein, SMAC-HA-ARL2 [43], that is strongly driven to the IMS by the 52-residue SMAC leader sequence prior to its cleavage, did not display any changes in HA staining intensity in response to growth in low serum. Thus, it is not a change in the quaternary structure of mitochondrial ARL2 that occurs in response to stress but rather a change in the amount of ARL2 at that compartment. We performed a few experiments using a mouse monoclonal antibody to ARL2, in contrast to the widespread use of our rabbit polyclonal antibody, and found that they both yielded the same results. The epitopes are not known for either of these antibodies but as they were independently derived they further strengthen our conclusions. Finally, we believe the increases in ARL2 are inside mitochondria and not on the outer membrane for two reasons: (1) we have recently shown that ARL2 acts from the IMS to regulate fusion (Newman et al., submitted), and (2) the increased staining in response to 2-deoxyglucose seen in HeLa cells resulted in an increased signal in ARL2 puncta localized at mitochondria when visualized by structured illumination microscopy (Fig 10E). Taken together, these results suggest that changes in ARL2, and by extension ELMOD2, staining is the result of changes in protein abundance specifically at this one organelle. These results cannot distinguish between an increase in protein abundance in mitochondria resulting from increased import vs decreased export or degradation, though we currently favor the former model. Because no correlative changes were observed for any other mitochondrial proteins tested, we conclude that ARL2 and ELMOD2 are changing in a highly specific and regulated fashion in response to specific cellular demands and signals; classic hallmarks of regulatory GTPase pathways.

ARL2 and ELMOD2 levels at mitochondria are sensitive to the presence of fusion-competent MFN2. This is in marked contrast to results with ARL3, the closest ARL2 paralog, ARFs or other family members and speaks to the specificity of these observations. For example, while ARF1 has been implicated in mitochondrial morphology [49] there is currently no evidence of its specific localization or direct actions at the organelle. MEFs deleted for MFN2 showed increased ARL2 and ELMOD2 staining at mitochondria, compared to controls. This increased staining was not due to the fragmentation observed in these MEFs, as *mfn1*^*-/-*^ MEFs did not show increased ARL2/ELMOD2 staining. Though MFN1 and MFN2 share ~60% identity [50] and a level of functional redundancy regarding fusion, MFN2 is proposed to have additional metabolic roles [7, 51–54] and may play a role in mitochondria/ER tethering [55], though this is contested [56, 57]. Increased levels of ARL2 and ELMOD2, along with mitochondrial fragmentation, could be reversed by expression of MFN2-myc in either *mfn2*^*-/-*^ or *mfn1*^*-/-*^*mfn2*^*-/-*^ MEFs. Importantly, expression of MFN1-myc could reverse the fragmentation but not the elevation in ARL2 and ELMOD2 levels. This demonstrates that these changes in protein levels were not strictly correlated with the loss of fusion, *per se*, but more linked to the function(s) of MFN2. A fusion dead mutant (MFN2[K109A]), which cannot reverse fragmentation in *mfn1*^*-/-*^*mfn2*^*-/-*^ MEFs [34], also failed to decrease ARL2/ELMOD2 levels in these cells. Perhaps the most telling linkage between ARL2/ELMOD2 levels and MFN2 fusogenic activity was found when MFN2[K109A] was expressed in *mfn2*^*-/-*^ MEFs. In this case fragmentation was partially reversed, presumably a result of the presence of MFN1 that can hetero-oligomerize with the mutant MFN2 [6], as were the elevation in ARL2 and ELMOD2 at mitochondria. And there was a correlation between cells in which fusion had been restored and staining had been reversed.

Results described here also further support our earlier conclusion that ARL2 acts in at least two distinct pathways in mitochondria, one affecting ATP levels that is independent of ELMOD2 and another that involves ELMOD2 and regulates mitochondrial fusion and motility. ARL2 siRNA results in ~50% loss in cellular ATP, while ELMOD2 knockdown had no effect on ATP levels [25]. Yet knockdown of either protein caused mitochondrial fragmentation and perinuclear clustering, presumably from the loss of plus end directed motility. Despite its discovery as an ARL2 GAP, we view ELMOD2 as likely acting in mitochondria as an effector, *i*.*e*., binding directly to the activated (GTP-bound) form of the GTPase and mediating aspects of its biological response, in this case fusion and motility. This is consistent with every other known ARF family GAP, each of which have been found to have effector properties [58–61]. Note that ARL2 and ELMOD2 behave the same in response to the loss of MFNs in MEFs (summarized in Table 1), in which mitochondrial fusion is altered. Yet the stressors of energy metabolism yield clear differences in responses of ARL2 and ELMOD2 (summarized in Table 2). Treatments that lower cellular ATP (2-deoxyglucose, oligomycin, antimycin A, etc.) do not alter ELMOD2 mitochondrial staining, consistent with ELMOD2 not playing a role in regulation of ATP. However, a subset of conditions (0 glucose, low serum, and galactose) that increase mitochondrial staining of ARL2 also increase that of ELMOD2. These stressors led to mitochondrial elongation, consistent with changes in ARL2 and ELMOD2 being linked to mitochondrial fusion. Work from others has shown that mitochondria fuse during stress, and that this pathway (called stress induced mitochondrial hyperfusion, or SIMH) requires MFN1 and not MFN2 [9–12]. Growth conditions (0 glucose, low serum) that led to increased ARL2/ELMOD2 and mitochondrial elongation promoted elongation in *mfn2*^*-/-*^ but not *mfn1*^*-/-*^ MEFs, consistent with previously reported data regarding SIMH [9]. Interestingly, increased ARL2/ELMOD2 occurred with stress in *mfn2*^*-/-*^ but not *mfn1*^*-/-*^ MEFs (Fig 8). Therefore, ARL2 and ELMOD2 may be acting upstream of MFN1 during SIMH. Based on these data and our rescue experiments with MFN2-myc, we speculate that increased MFN1 activity during SIMH may lead to increased ARL2/ELMOD2 in mitochondria, and that increased MFN2 activity may lead to decreased ARL2/ELMOD2.

The simple observations that the amounts of ARL2 and ELMOD2 at mitochondria are sensitive to cell attachment and density suggest that a signaling pathway exists to communicate information from the cell surface to mitochondria. A precedent might be seen in STAT3, best known for its role in conveying signals from the cell surface to the nucleus but more recently shown to act also inside mitochondria [62–64]. We have identified a number of conditions and reagents that activate it and thus provide the tools and assays required to further explore mechanisms.

To summarize, we conclude that ARL2 and ELMOD2 are increased at mitochondria in response to several types of cellular stress, and that this increase is correlated with MFN2 activity and fusion. These data are highly supportive of other studies (Newman, et al., submitted) in which we show that ARL2 acts from the IMS to regulate fusion upstream of the MFNs. They are also consistent with data from the *ant1*^*-/-*^ mice, which showed that changes in ARL2 levels in mitochondria may be tissue specific as they were dramatically increased only in affected tissues, skeletal muscle and heart [30]. Together they support the model that ARL2 and ELMOD2 levels at mitochondria are regulated and responsive to stressors and changes in MFN2 fusogenic potential. The model that an ancient, highly conserved, and ubiquitous regulatory GTPase regulates essential aspects of mitochondrial functions, morphology, and motility has broad significance and potential impact to our understanding of the basic cell biology of this organelle and its roles in human disease.

## Materials and methods

### Antibodies & reagents

Rabbit polyclonal antibodies directed against human ARL2, ARL3, and ELMOD2 were generated in our lab and have been described previously [25, 30, 47]. Characterization of the specificity of these reagents has included comparisons to pre-immune serum and antigen competition in both immunoblots and indirect immunofluorescence. Each of these methods has been optimized for specificity and sensitivity of these antibodies. The following antibodies were obtained from commercial sources: HA (Covance MMS-101P), HSP60 (Enzo Life Sciences ADI-SPA-807), cytochrome c (BD Biosciences 556432), myc (Invitrogen R950-25), TOM20 (BD Biosciences 61228), OPA1 (BD Biosciences 612606), Complex V subunit alpha (Mitosciences MS502), NDUFA9 (Mitosciences MS111), UQCRC2 (Mitosciences MS304), alpha tubulin (Sigma T9026), ELMOD2 (St. John’s Laboratory STG27186). We used a commercial ELMOD2 antibody for western blotting because it generated immunoblots with fewer nonspecific bands (as determined by antigen competition). The following antibody dilutions were used in our studies for immunofluorescence: ARL2 (1:2000), ELMOD2 (1:500), ARL3 (1:1000), HSP60 (1:5000), myc (1:1000), HA (1:2000), cytochrome c (1:2000), TOM20 (1:5000), OPA1 (1:100), Complex V subunit alpha (1:200), NDUFA9 (1:200), UQCRC2 (1:2000). The following antibody dilutions were used for immunoblotting: ARL2 (1:2000), ELMOD2 (1:500), OPA1 (1:1000), alpha tubulin (1:2500), myc (1:1000).

### Cloning and constructs

The following plasmids were generously provided by David Chan: MFN1-10xmyc and MFN2-16xmyc in pcDNA3.1, and MFN2 CMT2A mutations [V69F], [L76P], [R94Q], and [R274Q] in MFN2-7xmyc in pcDNA3.1 [6, 34]. The MFN2[K109A]-16xmyc plasmid (in pcDNA3.1) was generated in the lab of David Chan and obtained through Addgene (plasmid 26051). CMVΔ6 SMAC-HA-ARL2 and OCT-HA-ARL2 have been described and characterized previously [43].

### Cell culture

Human cervical carcinoma (HeLa), mouse embryonic fibroblast (MEF) and African green monkey kidney (COS7) cell lines were used in our studies to allow comparisons between them, ensure against a phenomenon that may be unique to one line, and to allow comparisons between cells displaying different mitochondrial morphologies. All were used as they are relatively flat, making them good for imaging. Additionally, we routinely obtain high transfection efficiencies (50–90%) and can readily detect endogenous ARL2 and ELMOD2 in HeLa and COS7 cell lines. Most experiments described here were performed in each of the different cell lines and we obtained very similar results. Cells were grown in DMEM medium, supplemented with 10% fetal bovine serum (FBS; cat# 11965–092, Invitrogen, Carlsbad, CA) and 2 mM glutamine at 37°C in the presence of 5% CO_2_. For imaging, cells were grown on matrigel (BD Biosciences #356231) coated coverslips. HeLa and COS7 cells were originally obtained from the ATCC. Immortalized MEFs from wild type, *mfn1*^*-/-*^, *mfn2*^*-/-*^, or *mfn1*^*-/-*^*mfn2*^*-/-*^, mice were a generous gift from Dr. David Chan [6, 7, 32, 33].

### Treatment with metabolic stressors

Prior to exposure to stressors, cells were plated on matrigel-coated coverslips and allowed to attach for at least four hours in standard DMEM medium. For growth in medium containing different carbon sources, the medium was exchanged after cells had attached. Cells were then fixed 24 or 48 hours later. Our normal DMEM medium contains 25 mM glucose supplemented with 10% FBS. No glucose medium is DMEM with no added glucose (cat# 11966–025, Invitrogen) supplemented with 10% FBS. Low serum medium is DMEM (25 mM glucose) supplemented with 2% FBS. Galactose medium was previously described [39] and was made with DMEM containing no glucose but with 10 mM galactose, 10 mM HEPES pH 7.4, and 10% FBS.

For other treatments, cells were plated and maintained in standard (25 mM glucose) DMEM with 10% FBS. One day after plating, cells were treated with the following drugs for the following times: 25 mM 2-deoxyglucose (Sigma cat#D8375, 16 hours), 10 μM oligomycin (Seahorse cat#101706–100, 16 hours), 10 μM antimycin A (Seahorse cat#101706–100, 16 hours), 10 μM rotenone (Seahorse cat#101706–100, 16 hours).

### Mitochondrial fractionation

Mitochondria were fractionated using a previously published protocol [65]. Cells were washed twice in PBS, incubated for 10 minutes in 5 mM EDTA in PBS, and collected. Cells were pelleted and washed in TD buffer (25 mM Tris-HCl, pH 7.4, 0.7 mM Na_2_HPO_4_, 133 mM NaCl, 5 mM KCl). Cells were resuspended in RSB buffer (10 mM Tris-HCl pH 7.4, 10 mM NaCl, 1.5 mM CaCl_2_, protease inhibitors (Sigma #P-2714), to a final volume of about 10x the volume of the cell pellet, and incubated on ice for 20 minutes prior to homogenization with a Dounce glass/glass tight homogenizer (30 strokes). Lysis of cells was verified using trypan blue. MS buffer, prepared as a 2.5X stock, was added to yield a final concentration of 1x MS (5 mM Tris-HCl pH 7.4, 210 mM mannitol, 70 mM sucrose, 5 mM EDTA pH 8). Unbroken cells and nuclei were removed by centrifugation (10 min., 1000xg), after which mitochondria were pelleted from the post-nuclear supernatant (20 min., 14,000xg). Mitochondria were then solubilized for 30 minutes on ice in 1% CHAPS, 25 mM HEPES pH 7.4, 100 mM NaCl, and protease inhibitors.

### Western blotting

Cells were harvested by rinsing twice with PBS, collected by incubation in 5 mM EDTA in phosphate buffered saline (PBS; 140 mM NaCl, 3 mM KCl, 10 mM Na_2_HPO_4_, 2 mM KH_2_PO_4_, pH 6.75), and pelleted in a microfuge (14,000 rpm, 4°C). Cells were lysed in 1% CHAPS, 25 mM HEPES pH 7.4, 100 mM NaCl, and protease inhibitors (Sigma #P-2714) on ice for 30 minutes, and the S14 was obtained by clarifying lysates by centrifugation for 30 minutes (14,000 rpm, 4°C). Protein concentrations were determined by Bradford Assay (Bio-Rad) using bovine serum albumin as standard. Samples were separated on 13% polyacrylamide gels and wet-transferred to nitrocellulose membranes (Bio-Rad #162–0112) at 70V for 2.5 hours. Western blotting procedures were carried out at room temperature. Membranes were blocked in blotto (5% (w/v) dry milk, 50 mM Tris pH 8, 2 mM CaCl_2_, 80 mM NaCl, 0.2% (v/v) Tween-20, 0.02% sodium azide) for 1 hour. When probing for ARL2, membranes were blocked in an alternate blocking buffer (10% goat serum, 5% Tween-20 in PBS), freshly filtered through a 0.2 μm membrane. Membranes were then incubated with primary antibody in blocking buffer at 4°C overnight. Removal of excess primary antibody was carried out by washing the membranes in PBST (PBS with 0.1% Tween-20) three times for 10 min each. HRP-conjugated secondary antibodies (GE cat #NA934V, #NA931V) were diluted 1:5,000 in PBST and incubated with the membrane for 1 hour at room temperature. Excess secondary antibody was removed by washing the membranes in PBST 3 times for 10 min each, then incubated in luminol containing solution (0.1 mM Tris-HCl pH 8.0, 1.2 mM luminol, 0.2 mM p-coumaric acid, 0.009% hydrogen peroxide) for 1 min prior to exposure to film.

### Transfections

Cells at 90% density or higher were transfected in 6 well plates using the following protocols. Both the amount of DNA and the ratio of Lipofectamine: DNA were separately optimized for ARL2 expression in HeLa and COS7 cells. A ratio of 2 μg Lipofectamine: 1 μg DNA yielded the highest transfection efficiency. ARL2 plasmids (2 μg) were diluted in 250 μL Optimem (Invitrogen). Lipofectamine 2000 (4 μg; Invitrogen) was diluted in a separate tube containing 250 μL Optimem, vortexed briefly, and incubated at room temperature for 5 minutes. The tubes were mixed and incubated 20 minutes. Cell culture medium was changed to 1.5 ml of Optimem, and transfection complexes (500 μL) were added dropwise to the cells. After 4 hours, cells were trypsinized and replated onto coverslips, typically at a 1:3 (24 hour time point) or 1:4 (48 hour time point) split. This transfection typically resulted in at least 70% of cells overexpressing ARL2.

MEFs were transfected using a similar protocol. Both the amount of DNA and the ratio of Lipofectamine: DNA were optimized for expression in MEFs. A ratio of 3 μg Lipofectamine: 1 μg DNA yielded the highest percentage of transfected cells, based upon immunofluorescent staining. Plasmids (5 μg) and Lipofectamine 2000 (15 μg; Invitrogen) were diluted in separate tubes, each containing 250 μL Optimem. The tube containing Lipofectamine was vortexed briefly, and incubated at room temperature for 5 minutes. The tubes were then mixed and incubated 20 minutes at room temperature. Cell culture medium was changed to 1.5 ml of Optimem, and transfection complexes (500 μL) were added dropwise to the cells. After 4 hours, cells were trypsinized and replated onto matrigel-coated coverslips, typically at a 1:4 split, and allowed to attach overnight. This transfection protocol typically resulted in ~40% of MEFs expressing MFN1-myc or MFN2-myc.

### Immunofluorescence

Cells grown on matrigel-coated coverslips were fixed in a pre-warmed (37°C) solution of 4% paraformaldehyde in PBS (10 mM Na_2_HPO_4_, 2 mM KH_2_PO_4_, pH 6.75, 140 mM NaCl, 3 mM KCl) for 15 minutes at room temperature, and then permeabilized with 0.1% (v/v) Triton X-100 in PBS for 10 minutes at room temperature. Incubation with primary antibodies was carried out in filtered PBS containing 1% (w/v) BSA at 4°C overnight, followed by 4 x 5 minute washes in PBS. Secondary antibodies (1:500, Alexa fluorophores 488 and 546, Invitrogen) were incubated in PBS containing 1% BSA for 1 hour at room temperature. Secondary antibody was removed by 2 x 5 minute washes in PBS. DNA was then stained with Hoechst 33342 (1 μM) (excitation max = 350 nm, emission max = 461 nm) for 4 minutes, followed by 2 x 5 minute washes in PBS. Coverslips were then mounted onto slides using Prolong Antifade (Invitrogen). To perform pre-permeabilization fixation, cells were treated with ice-cold 0.5% saponin in PBS for 1 minute on ice and immediately fixed with ice cold 4% PFA. Staining was then performed as described above. We routinely perform staining with only secondary antibody incubation, to ensure that the signal we observe is specific to our primary antibodies.

### Imaging

Images were acquired using an Olympus FV1000 microscope and Olympus Fluoview v1.7 software, using a 100x oil objective (1.45 NA). Alexa 488 (excitation maximum = 496 nm, emission maximum = 519 nm) and 546 (excitation maximum = 556 nm, emission maximum = 573 nm) fluorophores were imaged using 488 and 543 laser excitation, respectively. We ensured that the signal we observe while double labeling was not due to bleed through by labeling cells separately with each fluorophore and imaging with each laser line. Images were acquired with a resolution of 1024x1024 pixels and 16 bit depth. Z-stacks were acquired with a step size of 0.37 μm, which were converted to average image intensity projections using ImageJ, where indicated. Whenever ARL2 or ELMOD2 staining was compared between different experimental conditions, imaging settings were held constant between slides and conditions. Cells were plated using coverslips from the same batch, fixed the same day, stained with the same dilution of antibody at the same time, and imaged the same day using the same parameters (laser power, gain, pinhole, and offset). No adjustments to ARL2/ELMOD2 staining were made post-acquisition (other than increasing brightness and contrast of images for easier visualization, which was applied uniformly to all images in any comparison), and post-imaging processing of ARL2 and ELMOD2 staining was limited to generation of z-stack projections. We noted that z-stack projections of ELMOD2 stained cells resulted in higher quality images, so are used for ELMOD2 images. No such improvement was noted with z stack projections of ARL2 stained cells, so single focal planes are shown in order to minimize image processing. Focal planes selected for presentation were chosen with regard to bringing the most mitochondria in focus.

### Imaging quantification

Image quantification was performed using ImageJ. Individual cells were cropped from a field, and 8 bit average z stack projections were generated. Automatic thresholding was performed on the mitochondrial channel (HSP60 or cytochrome c) using Otsu’s method [66], to generate a mask. The mean pixel intensity in the ARL2 or ELMOD2 channel was then measured within the mask. For each condition, at least 10 cells were analyzed from one representative experiment, and each experiment quantified here has been repeated at least three times. Data represented are an average, normalized using the average of the control condition. Error bars represent standard error of the mean (SEM). To determine if increases in ARL2/ELMOD2 staining were significant, one tailed t tests were performed using Prism. One asterisk (*) denotes p<0.05, two (**) denotes p<0.01, three (***) denotes p<0.001, and four (****) denotes p<0.0001.

### Structured Illumination Microscopy (SIM)

Cells were imaged on a Nikon N-SIM using a 100X (NA 1.49) oil objective. For each cell, a widefield image was obtained in addition to raw SIM data. Reconstruction was achieved using the Nikon Elements software, and reconstruction parameters suggested by the software were adjusted with reference to the widefield image, to avoid previously described artifacts (http://www.gelifesciences.com/gehcls_images/GELS/Pdf%20documents/2015-05-06-Pellett-Bitesizebio-Seminar.pdf).

### Authentication of key reagents and data reproducibility

All cell lines used in our studies were originally obtained from ATCC, with the exception of mouse embryo fibroblast (MEF) lines that were either made by collaborators or colleagues who publish them and send them directly to us. All cell lines are routinely (approximately monthly) screened for the presence of mycoplasma, using DNA staining or PCR protocols. Records are maintained of cell line sources and passage number for each cryopreserved sample and no cells are used beyond passage 30. Every experiment was repeated at least twice, yielding similar results, and typically several more than two times. Because cell lines are clonal they are not viewed as biological replicates but technical ones when plated on multiple cover slips or dishes. The numbers of cells analyzed is stated for each condition, when quantification of pixel intensities was performed, as described above.

Antibodies used in these studies that were generated by contracts to companies for animal injections using our purified proteins (ARL2, ARL3, and ELMOD2) and characterized by us *at a minimum* for specificity in immunoblotting and immunofluorescence assays by comparison of immune to pre-immune sera and using (purified) antigen competition. When possible we also use antigen competition to confirm/test for specificity of antibodies obtained from other researchers or vendors. All antibodies are tested for recognition of a protein of the expected molecular weight in immunoblots. When an appropriate plasmid is available we also use transient over-expression in immunoblotting to confirm band with correct electrophoretic mobility.

## Abbreviations

ARL2: ADP-ribosylation factor-like 2
DRP1: dynamin related protein 1
ELMOD2: ELMO-domain containing protein 2
ETC: electron transport chain
FBS: fetal bovine serum
GAP: GTPase activating protein
IM: inner (mitochondrial) membrane
IMS: intermembrane space
MFN1/2: mitofusin 1/2
MLS: mitochondrial localization sequence
OCT: ornithine carbamoyltransferase (or the 32 residue leader sequence derived from the human protein)
OM: outer (mitochondrial) membrane
OPA1: optic atrophy 1
ROS: reactive oxygen species
SIMH: stress induced mitochondrial hyperfusion
SMAC: second mitochondria-derived activator of caspases, or the 59 residue leader sequence derived from the human protein; aka Diablo
